# Epidemic Cerebro-Spinal Meningitis, in Elmira, Steuben Co., N. Y.

**Published:** 1857-04

**Authors:** Frank H. Hamilton


*Epidemic Cerebro-spinal Meningitis, in Elmira, Steuben Co., N. Y.*—Having occasion to visit Elmira, in Steuben Co., N. Y., during the last week, I was requested by Drs. Flood & Purdy, to see a case of epidemic cerebro-spinal meningitis.

I found a lad, about eight years old, apparently dying, after an illness of four days. He was lying with his head strongly drawn back, as in opisthotons; deglutition was difficult; the pupils of his eyes possessed their natural impressibility to light, but he was unable to speak. The functions of his mind seemed to be unimpaired. His pulse was rapid, but not so feeble as his dying appearance had led me to think I should find it; indeed the very intelligent gentlemen who were in attendance, said that this condition, or general appearance was sometimes present a long time before death. Upon his body were numerous petechiæ, now very much faded, but which were at first quite dark.

The father of this child had died a few days before, in the same manner, after an illness of forty-eight hours.

This epidemic commenced in February, during the prevalence of the south wind, and after the disappearance of the snow. Scarlatina, in a mild form, had preceded it as an epidemic. It was, thus far, confined to the valley in which the village of Elmira is situated, and had occurred mostly among the poor, yet not entirely. The family whose house I visited, was in a comfortable dwelling, and in easy circumstances. The soil of this valley is composed chiefly, I think, of sand and gravel, and allows the surface to become easily drained. It is probable, however, that a substratum of frost has not permitted this drainage to be complete during the period of the present epidemic.

In all, twenty cases had occurred, of which only four had recovered. It was not confined to any period of life.

The usual signs which characterize the disease, are a general malaise, coldness of the extremities, and of the face, nose, chin, ears, while the head is rather warmer than natural; stupor, occasionally delirium, and sometimes no cerebral disturbance whatever. The head is gradually drawn back, and the muscles of the neck become rigid. Often, no pulsation can be felt at the wrist, when the physician is first called. The stomach and bowels are not much, if at all disturbed. Petechiæ occur at an early period.

The plan of treatment adopted has generally been stimulating: such as alone seemed to be indicated. Hot baths, medicated with cayenne pepper, mustard, brandy, &c., also hot mustard cataplasms, and frictions. Internally the patients have taken brandy, camphor, quinine, &c.

Since I have returned home, Dr. Flood has informed we that fifteen more cases have occurred, of which four terminated fatally within from one to two hours after the accession of the disease; but that under the plan of treatment described, four others, who were seen within from twenty to thirty minutes after the attack, recovered.

FRANK H. HAMILTON.

